# A comparison of perioperative outcomes of Video-Assisted Thoracic Surgical (VATS) Lobectomy with open thoracotomy and lobectomy: Results of an analysis using propensity score based weighting

**DOI:** 10.1186/1750-1164-4-1

**Published:** 2010-03-22

**Authors:** Walter J Scott, Ronald S Matteotti, Brian L Egleston, Salewa Oseni, James F Flaherty

**Affiliations:** 1 Fox Chase Cancer Center, Philadelphia, Pennsylvania, USA

## Abstract

**Background:**

Randomized trials comparing VATS lobectomy to open lobectomy are of small size. We analyzed a case-control series using propensity score-weighting to adjust for important covariates in order to compare the clinical outcomes of the two techniques.

**Methods:**

We compared patients undergoing lobectomy for clinical stage I lung cancer (NSCLC) by either VATS or open (THOR) methods. Inverse probability of treatment weighted estimators, with weights derived from propensity scores, were used to adjust cohorts for determinants of perioperative morbidity and mortality including age, gender, preop FEV1, ASA class, and Charlson Comorbidity Index (CCI). Bootstrap methods provided standard errors. Endpoints were postoperative stay (LOS), chest tube duration, complications, and lymph node retrieval.

**Results:**

We analyzed 136 consecutive lobectomy patients. Operative mortality was 1/62 (1.6%) for THOR and 1/74 (1.4%) for VATS, P = 1.00. 5/74 (6.7%) VATS were converted to open procedures. Adjusted median LOS was 7 days (THOR) versus 4 days (VATS), P < 0.0001, HR = 0.33. Adjusted median chest tube duration (days) was 5 (THOR) versus 3 (VATS), P < 0.0001, HR = 0.42. Complication rates were 39% (THOR) versus 34% (VATS), P = 0.61. Adjusted mean number of lymph nodes dissected per patient was 18.1 (THOR) versus 14.8 (VATS), p = 0.17.

**Conclusions:**

After balancing covariates that affect morbidity, mortality and LOS in this case-control series using propensity-weighting, the results confirm that VATS lobectomy is associated with a statistically significant shorter LOS, similar mortality and complication rates and similar rates of lymph node removal in patients with clinical stage I NSCLC.

## Introduction

The routine use of video-assisted thoracic surgical (VATS) lobectomy for the treatment of resectable non-small cell lung cancer (NSCLC) remains controversial. Data supporting the use of VATS lobectomy come from randomized trials [1-4], a multicenter phase II study [5], case-control series and large retrospective series [6]. Meta-analyses have recently been published [7,8]. The randomized trials enrolled relatively small numbers of patients and retrospective case series are subject to selection biases. Other recent publications have been case-control series [9,10].

Most case-control techniques attempt to decrease the effect of selection bias when comparing two non-randomized treatment groups by analyzing patients that are "matched" based on preoperative variables that are known to affect the outcomes that are being studied. It can be difficult to find appropriate control patients for a case matching study unless a large control population is available. Even then, cases without an appropriate match in either the control or treatment groups are not usable for analysis. At times, eliminating the cases that cannot be matched may actually result in an increase in selection bias, casting doubt on the conclusion that differences in outcomes between the two groups are due to treatment effects [11].

Propensity score based weighting is a rigorous statistical technique for making nonrandomized comparisons that in theory allows all of the patient data to be used from two treatment groups. We used this method to adjust for selection differences between two patient populations with clinical stage I NSCLC who underwent lobectomy performed through either an open or VATS technique. We then compared the perioperative outcomes of the two adjusted groups.

## Methods

Approval for this retrospective data analysis of prospectively collected data was obtained from the Institutional Review Board. Patients with known or suspected clinical stage I NSCLC who underwent surgical resection at Fox Chase Cancer Center (FCCC) during two periods of time, from mid-2003 until November, 2005 and from November 2005-through 2008 were analyzed. Patients undergoing lobectomy, bilobectomy or segmentectomy were included. During the study periods, patients underwent standard preoperative staging with chest computed tomography (CT) and positron emission tomography with CT (PET/CT). At the time of the study, brain MR was performed in patients with neurologic symptoms or for patients with T2 tumors. Cervical mediastinoscopy was performed selectively for patients with abnormal lymph nodes on any imaging study, or for those with T2 or more central tumors.

All of the operations in this series were performed by a single surgeon (WJS). Standard anesthetic management with single lung ventilation including restriction of intraoperative fluids was used in all patients. Open thoracotomy (THOR) was most commonly performed through a posterolateral incision with entry into the chest through the fifth intercostal space. Resection of a small portion of the 6^th ^rib was routine. Muscle sparing incisions were used occasionally and rib resection was not performed in those instances. VATS lobectomy (VATS) was performed using three incisions, a 5 cm or smaller access thoracotomy and two additional 1 cm incisions. Rib spreading and rib resection were not performed. Visualization was achieved using the video thoracoscope.

Lymph node dissection was routinely performed in all cases (THOR or VATS). Postoperative pain control was achieved using patient-controlled analgesia delivered through thoracic epidural catheters (PCEA) or through the use of patient-controlled intravenous narcotics (PCA). Most recently, pain control for VATS lobectomies consists of PCA narcotics supplemented with a continuous local anesthetic infusion (0.2% ropivacaine) though subpleural catheters placed intraoperatively. Patients converted from VATS to thoracotomy were included in the VATS group. All complications were graded according to the National Cancer Institute Common Terminology Criteria for Adverse Events version 3.0 [12].

Patients undergoing thoracotomy (open cases) were the control population and patients undergoing VATS procedures comprised the treatment group. The baseline characteristics of the two groups are shown in table 1 (demographics). The endpoint of the study was postoperative length of stay (LOS), duration of chest tube placement, and overall complication rates. Because this was a nonrandomized comparison of two treatments, the two groups were adjusted for characteristics that were known to influence the main study outcomes. We chose age, sex, the preoperative FEV1 percent predicted and two additional measures, the Charlson Comorbidity Index (CCI) and the American Society of Anesthesiology (ASA) score. (Table 2) The CCI consists of the sum of weighted scores for 19 medical conditions that have been shown to affect mortality. The CCI has been validated in a surgical population of lung cancer patients [13]. The ASA score is a rating scale used by anesthesiologists to estimate the overall condition of a patient in the preoperative period Table 3[14].

**Table 1:** Charlson Comorbidity Index (CCI)

Score	Condition
1	Coronary artery disease^a^ Congestive heart failure Chronic pulmonary disease Peptic ulcer disease Peripheral vascular disease Mild liver disease Cerebrovascular disease Connective tissue disease Diabetes mellitus Dementia
2	Hemiplegia Moderate to severe renal disease Diabetes with end-organ damage Any prior tumor (within 5-years of diagnosis)^b^ Leukemia Lymphoma
3	Moderate to severe liver disease
6	Metastatic solid tumor AIDS (not only HIV positive)

Adapted from Birim O, EJCTS 2003 with permission

**Table 2:** Patient Characteristics, Unadjusted data, % or mean (SD)

	Thoracotomy n = 62	VATS n = 74	p-value
Male	47%	30%	0.054
FEV1% pred	79 (19)	85 (19)	0.040
CCI	1.7 (1.3)	1.7 (1.3)	0.982
Age	65 (10.3)	67 (10.9)	0.489
ASA	2.3 (0.6)	1.8 (0.7)	<0.001

**Table 3:** American Society of Anesthesiologist (ASA) Score

**Class 1**	Healthy patient, no medical problems
**Class 2**	Mild systemic disease
**Class 3**	Severe systemic disease, but not incapacitating
**Class 4**	Severe systemic disease that is a constant threat to life
**Class 5**	Moribund, not expected to live 24 hours irrespective of operation
An **e **is added to the status number to designate an emergency operation. An organ donor is usually designated as Class 6	

In order to adjust for baseline differences between those patients who did and did not have VATS, we used propensity score based adjustment through propensity score based weighting [15]. Similar propensity score based weighting has been used in a variety of settings to investigate treatment effects using observational data [16,17]. The propensity scores, which are the probabilities of receiving VATS given potential confounders of treatment assignment, were estimated by a multiple logistic regression. The adequacy of the propensity score model was verified by examining adjusted differences in potential confounder variables between the treatment groups. The lack of significant differences in propensity-score adjusted averages of the confounder variables suggested that they could not be confounders after adjustment.

We used Fisher's exact tests and T-tests to assess unadjusted differences. For inferences concerning length of stay and time until chest tube removal, we used Cox proportional hazards regressions weighted by the inverse of the probability of receiving the treatment actually received. The weight would be the inverse of the propensity score for those in the VATS group and the inverse of one minus the propensity score for those in the thoracotomy group. The bootstrap [18] with 2500 resamples was used to calculate the standard errors. Cox models were used for the time to event variables since some patients were lost to follow-up (for example, discharged with a chest tube) and hence had censored data. We used simple linear regressions similarly weighted by the inverse of the probability of receiving the treatment actually received for adjusted inferences concerning the number of lymph nodes removed and the number of lymph node stations sampled. We used propensity score based weighted logistic models for adjusted inferences concerning complication rate differences.

## Results

The study population consisted of 136 patients with clinical stage I NSCLC. There were 62 patients from 2003 through late-2005 who underwent lobectomy performed through an open thoracotomy and 74 patients who underwent VATS lobectomy from late 2005 through mid-2008. Patient demographics are shown in Table 1. There were more men in the group undergoing thoracotomy and the VATS group had slightly better pulmonary function based on preoperative FEV1 (% predicted). There were no significant differences between groups with respect to clinical stage, pathologic stage and histology (Table 4). There were no significant differences between groups with respect to the type of lobectomy performed (Table 4). Conversion of a VATS lobectomy to an open procedure occurred in 3/74 (4%) of patients. This generally involved enlargement of the access thoracotomy and placement of a small retractor, and then performance of the lobectomy with standard techniques. These patients were included in the VATS group for the purpose of analysis.

**Table 4:** Details of Surgical Procedures

	Open lobe (n = 62)		VATS lobe (n = 74)		P value
Histology					

Adeno	44	71%	50	68%	0.898

Squamous	9	15%	12	16%	

Other	9	15%	12	16%	

Clinical stage					

IA	49	79%	65	88%	0.242

IB	13	21%	9	12%	

Pathologic stage					

IA	32	52%	45	61%	0.165

IB	20	32%	12	16%	

II	5	8%	7	9%	

III, IV	5	8%	10	14%	


Type of resection					

RUL	20	32%	23	31%	0.168

RML	3	5%	10	14%	

RLL	8	13%	12	16%	

LUL	18	29%	14	19%	

LLL	10	16%	10	14%	

Bilobectomy	3	5%	1	1%	

Segmentectomy	0	0%	4	5%	

Adjusting for observable differences between cohorts and balancing important covariates using propensity-score weighting resulted in two well-matched groups for analysis (Table 5). The perioperative results and analyses were based on comparison of data from the adjusted groups. Patients undergoing VATS lobectomy had a shorter length of stay (VATS, 4 days versus THOR, 7 days, p < 0.001, HR = 0.33 in favor of VATS). Patients in the VATS group also had their chest tubes removed earlier (VATS, 3 days versus THOR, 5 days, p < 0.0001, HR = 0.42 in favor of VATS). The number of lymph node stations dissected in the VATS and THOR groups was similar (4.6 stations versus 4.3 stations, respectively, p = 0.31) as was the total number of lymph nodes examined in the specimen (18.1 versus 14.8, p = 0.17). Mortality rates were similar, with 1/74 (1.4%) in the VATS group and 1/62 (1.6%) in the THOR group (p = 1.00).

**Table 5:** Patient Characteristics Adjusted Data, N = 136

	Thoracotomy n = 62	VATS n = 74
Male	40%	39%
FEV1% pred	80 (19)	81 (20)
CCI	1.6 (1.3)	1.7 (1.3)
Age	66 (10)	66 (11)
ASA	2.1 (0.6)	2.1 (0.7)

Perioperative and postoperative complications were classified and graded using the National Cancer Institute Common Terminology Criteria for Adverse Events version 3.0 (Table 6). No differences in complication rates were noted for either cohort (VATS or THOR). No complications were recorded for 40/62 (65%) THOR patients compared to 52/74 (70%) of VATS patients (p = 0.61). There was a trend suggesting a decrease in pulmonary complications in the VATS group 10/74 (14%) compared to the THOR group 20/62 (32%) (p = 0.10). Continuous heart rate monitoring was routine during the second period of the study when VATS lobectomy was initiated. Therefore the low number of arrhythmias in the THOR patients may reflect the fact that only those arrhythmias that were persistent or were associated with symptoms were recorded for those patients, whereas even brief periods of atrial fibrillation were detected and recorded for the VATS group.

**Table 6:** Complication Rates

Complication	Open N = 62 Unadjusted data	VATS N = 74 Unadjusted data	P value (unadjusted)	P value (adjusted)
None	40 (65%)	52 (70%)	0.58	0.61

Pulmonary	20 (32%)	10 (14%)	0.01	0.10

Cardiac-arrhythmia	2 (3%)	10 (14%)	0.07	0.71

Hemorrhage	1 (2%)	4 (5%)	0.38	0.88

Other	1 (2%)	2 (3%)	1.00	0.98

When complications were examined by age (70 years and older){Table 7), the trend toward a decrease in pulmonary complications associated with VATS was present but still did not reach statistical significance, perhaps because of the small number of patients in each group.

**Table 7:** Complication rates in patients aged 70 and older

Complication	Open N = 22	VATS N = 30	P value (adjusted)
None	14 (64%)	21 (70%)	0.77

Pulmonary	7 (32%)	3 (10%)	0.08

Cardiac-arrhythmia	2 (9%)	5 (17%)	0.69

Hemorrhage	0 (0%)	1 (3%)	1.00

Other	0 (0%)	1 (3%)	1.00

## Discussion

We used propensity score based weighting to perform a non-randomized comparison of patients who underwent wither open thoracotomy and lobectomy or VATS lobectomy for clinical stage I NSCLC. The statistical method that we used generates an adjusted population of patients whose outcomes are slightly different that the observed data. For example, that is the reason that the complication rates in Table 8 (39% for THOR and 34% for VATS) are slightly different than the unadjusted complication rates in Table 6 (35% for THOR and 30% for VATS). The adjusted rates are based on a larger number of patients and therefore should be considered the most generalizable data. After balancing the two cohorts based on preoperative covariates known to influence perioperative outcomes using propensity score based weighting, we found that patients undergoing VATS lobectomy for clinical stage I NSCLC had their chest tubes removed earlier (adjusted median 3 days versus 5 days, p < 0.001) and also had a shorter postoperative length of stay (adjusted median 4 days versus 7 days, p < 0.001)) than patients undergoing open thoracotomy. This is consistent with what others have observed from case control series [9,10] and large retrospective case series [6]. Patients undergoing VATS lobectomy may experience less pain, have improved respiratory mechanics, and develop less of an inflammatory response compared to open thoracotomy, all of which combined may lead to a decreased postoperative length of stay.

**Table 8:** Perioperative Results, Adjusted Data

**Op**.	N	Peri-op mortality (%)	Length of Stay (Adjusted median days)	Chest tube removal (Adjusted median days)	Adverse Events (Adjusted % of patients with at least one)	Lymph Node Stations (Adjusted # sampled/patient)	Lymph Nodes (Adjusted mean # removed/patient)
Thor	62	1 (1.6%)	7	5	39%	4.6	18.1
VATS	74	1 (1.4%)	4	3	34%	4.3	14.8
		P = 1.00	HR = 0.33	HR = 0.42			
P-value			P < 0.0001	P = 0.0001	P = 0.61	P = 0.31	P = 0.17

Despite the marked reduction in postoperative length of stay, we found no statistically significant difference in overall complication rates or specific complication rates between VATS patients and THOR patients. One reason for this is that the number of episodes of atrial fibrillation in the THOR group was probably underestimated since routine use of postoperative heart rhythm monitoring was phased in during the first part of the study when the open lobectomy operations were performed. Therefore, it is likely that episodes of atrial fibrillation in this group were missed and that the true rate of atrial fibrillation in this group was much higher. This would have increased the overall and arrhythmia complication rates in the THOR group. Other studies have suggested that atrial fibrillation rates after VATS lobectomy are not different than after thoracotomy and lobectomy [8,9].

Mortality rates were not different between groups in our study. We did observe a trend (p = 0.10) toward a decrease in respiratory complication rates in the VATS patients, especially in the 70 year and older group, with the rates of respiratory complications decreasing from 32% in the open group to 10% in the VATS group (p = 0.08). A decrease in respiratory complications in older patients undergoing VATS lobectomy compared to THOR was observed by Cattaneo et al. [10]. It is possible that a statistically significant difference in respiratory complication rates would have been observed in our study if the sample size had been larger.

Regarding oncologic efficacy of VATS compared to THOR, we found that all patients underwent compete resections with negative margins. The number of lymph node stations that were dissected and the total number of lymph nodes retrieved were similar for each group. Continued follow up will be required to determine oncologic efficacy of VATS compared to THOR.

The limitations of this study include its retrospective nature, the fact that management changes may have occurred over the study period that were not appreciated or reflected in the analysis (such as the change to continuous heart rate monitoring or other changes in postoperative management), and the sample size. The fact that is not a multicenter study is mitigated by the fact that our conclusions, while analyzed somewhat differently, are similar to those of other authors. The strengths of the study include the fact that all cases were performed by a single surgeon, it is a consecutive case series, and that covariates were balanced between the two cohorts using sophisticated statistical analysis.

## Conclusion

In summary, we found that patients undergoing VATS lobectomy were able to have their chest tubes removed sooner and were discharged sooner after undergoing lobectomy for clinical stage I NSCLC than patients who underwent thoracotomy and lobectomy for the same indications. A trend toward a decrease in pulmonary complications in the VATS group was also observed, especially in patients 70 years of age or older. The use of propensity score-based weighting in this series and its application to larger databases will allow clinicians to draw stronger conclusions about the relative merits of VATS lobectomy versus open thoracotomy and lobectomy in the treatment of the patient with early stage lung cancer.

## Abbreviations

ASA: American Society of Anesthesiologists; CCI: Charlson comorbidity index; CT: computed tomography; CTC: common toxicity criteria; FCCC: Fox Chase Cancer Center; FEV1 forced expiratory volume at 1 second; HR: hazard ratio; LOS: length of stay; MR: magnetic resonance; NCI: national cancer institute; NSCLC: non-small cell lung cancer; PCA: patient-controlled analgesia; PCEA: patient-controlled epidural analgesia; PET/CT Positron emission tomography and computed tomography; THOR: open thoracotomy and lobectomy; VATS: video-assisted thoracic surgery.

## Competing interests

The authors declare that they have no competing interests.

## Authors' contributions

WS performed all surgeries and drafted the manuscript.  RM collected data and revised the manuscript in its final version.  SO and JFF provided research support and data collection. Oseni presented these data at the American College of Chest Physicians meeting in fall, 2008. BLE is responsible for the statistical analysis of these data.  All authors have read and approved the final manuscript.
